# COVID-19 in a Long-Term Care Facility — King County, Washington, February 27–March 9, 2020

**DOI:** 10.15585/mmwr.mm6912e1

**Published:** 2020-03-27

**Authors:** Temet M. McMichael, Shauna Clark, Sargis Pogosjans, Meagan Kay, James Lewis, Atar Baer, Vance Kawakami, Margaret D. Lukoff, Jessica Ferro, Claire Brostrom-Smith, Francis X. Riedo, Denny Russell, Brian Hiatt, Patricia Montgomery, Agam K. Rao, Dustin W. Currie, Eric J. Chow, Farrell Tobolowsky, Ana C. Bardossy, Lisa P. Oakley, Jesica R. Jacobs, Noah G. Schwartz, Nimalie Stone, Sujan C. Reddy, John A. Jernigan, Margaret A. Honein, Thomas A. Clark, Jeffrey S. Duchin, Public Health – Seattle & King County, EvergreenHealth, and CDC COVID-19 Investigation Team, Public Health – Seattle & King County, EvergreenHealth, and CDC COVID-19 Investigation Team, Meaghan S. Fagalde, Jennifer L. Lenahan, Emily B. Maier, Kaitlyn J. Sykes, Grace Hatt, Holly Whitney, Melinda Huntington-Frazier, Elysia Gonzales, Laura A. Mummert, Hal Garcia Smith, Steve Stearns, Eileen Benoliel, Shelly McKeirnan, Jennifer L. Morgan, Daniel Smith, Michaela Hope, Noel Hatley, Leslie M. Barnard, Leilani Schwarcz, Nicole Yarid, Eric Yim, Sandra Kreider, Dawn Barr, Nancy Wilde, Courtney Dorman, Airin Lam, Jeanette Harris, Hollianne Bruce, Christopher Spitters, Snohomish Health District, Rachael Zacks, Jonathan Dyal, Michael Hughes, Christina Carlson, Barbara Cooper, Michelle Banks, Heather McLaughlin, Arun Balajee, Christine Olson, Suzanne Zane, Hammad Ali, Jessica Healy, Kristine Schmit, Kevin Spicer, Zeshan Chisty, Sukarma Tanwar, Joanne Taylor, Leisha Nolen, Jeneita Bell, Kelly Hatfield, Melissa Arons, Anne Kimball, Allison James, Mark Methner, Joshua Harney

**Affiliations:** ^1^Public Health – Seattle & King County; ^2^Epidemic Intelligence Service, CDC; ^3^CDC COVID-19 Emergency Response; ^4^Evergreen Health, Kirkland, Washington; ^5^Washington State Public Health Laboratory; ^6^Washington State Department of Health; ^7^Laboratory Leadership Service, CDC.; Seattle & King County; King County Medical Examiner’s Office; EvergreenHealth; CDC.

On February 28, 2020, a case of coronavirus disease (COVID-19) was identified in a woman resident of a long-term care skilled nursing facility (facility A) in King County, Washington.* Epidemiologic investigation of facility A identified 129 cases of COVID-19 associated with facility A, including 81 of the residents, 34 staff members, and 14 visitors; 23 persons died. Limitations in effective infection control and prevention and staff members working in multiple facilities contributed to intra- and interfacility spread. COVID-19 can spread rapidly in long-term residential care facilities, and persons with chronic underlying medical conditions are at greater risk for COVID-19–associated severe disease and death. Long-term care facilities should take proactive steps to protect the health of residents and preserve the health care workforce by identifying and excluding potentially infected staff members and visitors, ensuring early recognition of potentially infected patients, and implementing appropriate infection control measures.

On February 27, Public Health – Seattle and King County (PHSKC) was notified by a local health care provider of a patient whose symptom history and clinical presentation met the revised testing criteria^†^ for COVID-19, which included testing of persons with severe respiratory illness of unknown etiology (*1*). The patient was a woman aged 73 years with a history of coronary artery disease, insulin-dependent type II diabetes mellitus, obesity, chronic kidney disease, hypertension, and congestive heart failure, who resided in facility A along with approximately 130 residents who were cared for by 170 health care personnel. Beginning in mid-February, the facility had experienced a cluster of febrile respiratory illnesses. Rapid influenza test results were obtained from several residents; all were negative. The patient had cough, fever, and shortness of breath requiring oxygen for 5 days at facility A. She reported no travel or known contact with anyone with COVID-19. On February 24, she was transported to a local hospital because of worsening respiratory symptoms and hypoxemia.

Upon hospital admission, the patient was febrile to 103.3°F (39.6°C), tachycardic, and was found to have hypoxemic respiratory failure. On February 25, she required intubation and mechanical ventilation. Computed tomography scan showed diffuse bilateral infiltrates; however, multiplex viral respiratory panel and bacterial cultures of sputum and bronchoalveolar lavage fluid were negative. Four days after hospital admission, nasopharyngeal and oropharyngeal swabs and sputum specimens were collected to test for SARS-CoV-2; results were reported positive for all specimens on February 28. The patient died on March 2.

Following notification of the index case of COVID-19, PHSKC and CDC immediately began investigating the cluster of respiratory illness in facility A to collect information on symptoms, severity, comorbidities, travel history, and close contacts to known COVID-19 cases by interviewing patients or a proxy for cases in which the patient could not be interviewed. Diagnostic testing by real-time reverse transcription–polymerase chain reaction (RT-PCR) (*2*–*5*) was performed for patients and staff members meeting clinical case criteria for COVID-19 (*1*). As of March 9, a total of 129 COVID-19 cases were confirmed among facility residents (81 of approximately 130), staff members, including health care personnel (34), and visitors (14). Health care personnel with confirmed COVID-19 included the following occupations: physical therapist, occupational therapist assistant, environmental care worker, nurse, certified nursing assistant, health information officer, physician, and case manager. Overall, 111 (86%) cases occurred among residents of King County (81 facility A residents, 17 staff members, and 13 visitors) and 18 (14%) among residents of Snohomish County (directly north of King County) (17 staff members and one visitor).

Reported symptom onset dates for facility residents and staff members ranged from February 16 to March 5. The median patient age was 81 years (range = 54–100 years) among facility residents, 42.5 years (range = 22–79 years) among staff members, and 62.5 years (range = 52–88 years) among visitors; 84 (65.1%) patients were women (Table). Overall, 56.8% of facility A residents, 35.7% of visitors, and 5.9% of staff members with COVID-19 were hospitalized. Preliminary case fatality rates among residents and visitors as of March 9 were 27.2% and 7.1%, respectively; no deaths occurred among staff members. The most common chronic underlying conditions among facility residents were hypertension (69.1%), cardiac disease (56.8%), renal disease (43.2%), diabetes (37.0%), obesity (33.3%), and pulmonary disease (32.1%). Six residents and one visitor had hypertension as their only chronic underlying condition.

**TABLE:** Characteristics of patients with COVID-19 epidemiologically linked to facility A among residents of King and Snohomish counties — Washington, February 27–March 9, 2020

Characteristics	No. (%)			
	Resident (n = 81)	Health care personnel (n = 34)	Visitor (n = 14)	Total (n = 129)
Median age, yrs (range)	81 (54–100)	42.5 (22–79)	62.5 (52–88)	**71 (22–100)**
**Sex**				
Men	28 (34.6)	7 (20.6)	10 (71.4)	**45 (34.9)**
Women	53 (65.4)	27 (79.4)	4 (28.6)	**84 (65.1)**
**Hospitalized**				
Yes	46 (56.8)	2 (5.9)	5 (35.7)	**53 (41.1)**
No	3 (3.7)	30 (88.2)	9 (64.3)	**42 (32.6)**
Unknown	32 (39.5)	2 (5.9)	0	**34 (26.4)**
**Died**				
Yes	22 (27.2)	0	1 (7.1)	**23 (17.8)**
No	59 (72.8)	34 (100.0)	13 (92.9)	**106 (82.2)**
Chronic underlying conditions*^,†^				
Hypertension^§^	56 (69.1)	0	2 (14.3)	**58 (45.0)**
Cardiac disease	46 (56.8)	3 (8.8)	2 (14.3)	**51 (39.5)**
Renal disease	35 (43.2)	0	1 (7.1)	**36 (27.9)**
Diabetes mellitus	30 (37.0)	3 (8.8)	1 (7.1)	**34 (26.4)**
Obesity	27 (33.3)	0	3 (21.4)	**30 (23.3)**
Pulmonary disease	26 (32.1)	2 (5.9)	2 (14.3)	**30 (23.3)**
Malignancy	11 (13.6)	0	0	**11 (8.5)**
Immunocompromised	8 (9.9)	0	0	**8 (6.2)**
Liver disease	5 (6.2)	0	0	**5 (3.9)**

* Percentages represent the number with information on the comorbidity, irrespective of missing data.

^†^ Data on chronic underlying conditions were missing for four health care personnel and two visitors with COVID-19.

^§^ Hypertension was the only reported chronic underlying condition for 6 residents and 1 visitor with COVID-19.

As part of the response effort, approximately 100 long-term care facilities in King County were contacted through an emailed survey using REDCap (*6*), and information was requested about residents or staff members known to have COVID-19 or clusters of respiratory illness among residents and staff members. In addition, countywide databases of emergency medical service transfers from long-term care facilities to acute care facilities were reviewed daily for evidence of cases or clusters of serious respiratory illness. Routine active surveillance reports to PHSKC for influenza-like illness clusters from long-term care facilities were employed to identify clusters of illness consistent with COVID-19. All long-term care facilities with evidence of a cluster of respiratory illness were contacted by telephone for additional information, including infection control strategies in place and availability of personal protective equipment (PPE). Based on this information, the long-term care facilities were prioritized by risk for COVID-19 introduction and spread, and highest priority facilities were visited by response personnel for provision of emergency on-site testing and infection control assessment, support, and training. As of March 9, at least eight other King County skilled nursing and assisted living facilities had reported one or more confirmed COVID-19 cases.

Information received from the survey and on-site visits identified factors that likely contributed to the vulnerability of these facilities, including 1) staff members who worked while symptomatic; 2) staff members who worked in more than one facility; 3) inadequate familiarity and adherence to standard, droplet, and contact precautions and eye protection recommendations; 4) challenges to implementing infection control practices including inadequate supplies of PPE and other items (e.g., alcohol-based hand sanitizer)^ §^; 5) delayed recognition of cases because of low index of suspicion, limited testing availability, and difficulty identifying persons with COVID-19 based on signs and symptoms alone.

## Discussion

These findings demonstrate that outbreaks of COVID-19 in long-term care facilities can have a critical impact on vulnerable older adults. In Washington, local and state authorities implemented comprehensive prevention measures for long-term care facilities (*7*–*9*) that included 1) implementation of symptom screening and restriction policies for visitors and nonessential personnel; 2) active screening of health care personnel, including measurement and documentation of body temperature and ascertainment of respiratory symptoms to identify and exclude symptomatic workers; 3) symptom monitoring of residents; 4) social distancing, including restricting resident movement and group activities; 5) staff training on infection control and PPE use; and 6) establishment of plans to address local PPE shortages, including county and state coordination of supply chains and stockpile releases to meet needs. These strategies require coordination and support from public health authorities, partnering health care systems, regulatory agencies, and their respective governing bodies (*8*–*10*).

The findings in this report suggest that once COVID-19 has been introduced into a long-term care facility, it has the potential to result in high attack rates among residents, staff members, and visitors. In the context of rapidly escalating COVID-19 outbreaks in much of the United States, it is critical that long-term care facilities implement active measures to prevent introduction of COVID-19. Measures to consider include identifying and excluding symptomatic staff members, restricting visitation except in compassionate care situations, and strengthening infection prevention and control guidance and adherence (*7*,*9*,*10*).^¶^ Substantial morbidity and mortality might be averted if all long-term care facilities take steps now to prevent exposure of their residents to COVID-19. The underlying health conditions and advanced age of many long-term care facility residents and the shared location of patients in one facility places these persons at risk for severe morbidity and death. Rapid and sustained public health interventions focusing on surveillance, infection control, and mitigation efforts are resource-intensive but are critical to curtailing COVID-19 transmission and decreasing the impact on vulnerable populations, such as residents of long-term care facilities, and the community at large. As this pandemic expands, continued implementation of public health measures targeting vulnerable populations such as residents of long-term care facilities (*8*) and health care personnel will be critical. As public health measures are continually implemented, public information needs will only grow. To provide information for patients and families as well as communicate more broadly to all stakeholders, public officials and other community leaders need to work together to encourage everyone to understand and adhere to recommended guidelines to manage this outbreak.

**Summary**

**What is already known about this topic?**

Coronavirus disease (COVID-19) can cause severe illness and death, particularly among older adults with chronic health conditions.

**What is added by this report?**

Introduction of COVID-19 into a long-term residential care facility in Washington resulted in cases among 81 residents, 34 staff members, and 14 visitors; 23 persons died. Limitations in effective infection control and prevention and staff members working in multiple facilities contributed to intra- and interfacility spread.

**What are the implications for public health practice?**

Long-term care facilities should take proactive steps to protect the health of residents and preserve the health care workforce by identifying and excluding potentially infected staff members, restricting visitation except in compassionate care situations, ensuring early recognition of potentially infected patients, and implementing appropriate infection control measures.

## Footnotes

† https://emergency.cdc.gov/han/2020/han00428.asp.

§ Some examples of specific PPE challenges included initial lack of access to eye protection, frequent changing of PPE types as supply chains were disrupted and PPE was provided via various donations or supplies, and a need for ongoing auditing of PPE use to ensure consistent and safe use of PPE by staff members (e.g., not touching or adjusting face protection, primarily facemasks, during extended use).

¶ https://www.doh.wa.gov/Portals/1/Documents/1600/coronavirus/RecommendationsForLTC-COVID19.pdf.
